# Comparison of three measures for insomnia in ischemic stroke patients: Pittsburgh sleep quality index, insomnia severity index, and Athens insomnia scale

**DOI:** 10.3389/fneur.2023.1118322

**Published:** 2023-08-30

**Authors:** Shuzhen Niu, Qian Wu, Silian Ding, Lingchun Wu, Li Wang, Yan Shi

**Affiliations:** ^1^Shanghai Tenth People's Hospital, School of Medicine, Tongji University, Shanghai, China; ^2^Shanghai Fourth People's Hospital, Shanghai, China; ^3^Department of Nephrology, Shanghai Zhabei Central Hospital, Shanghai, China

**Keywords:** ischemic stroke, insomnia, diagnosis, Pittsburgh sleep quality index (PSQI), insomnia severity index (ISI), Athens insomnia scale (AIS)

## Abstract

**Objective:**

This study investigated the consistency and determined the optimal threshold values of three scales in the diagnosis of insomnia of ischemic stroke (IS) patients.

**Methods:**

Participants in this study consisted of 569 acute IS patients. All 569 patients completed the assessment of the three insomnia scales. Insomnia of IS patients were assessed by Pittsburgh sleep quality index (PSQI), Insomnia Severity Index (ISI), and Athens insomnia scale (AIS). Also, basic patient information, neurological function, and activities of daily living were assessed. General information was compared between the insomnia group and the no-insomnia group. Cronbach’s α coefficients, Cohen’s Kappa consistency, Receiver operating characteristic (ROC) curve and DeLong’s test analysis were used to analyze the reliability and diagnostic validity of PSQI, ISI, and AIS.

**Results:**

The PSQI and ISI showed high reliability with Cronbach’s α of 0.875 and 0.858, respectively, while the AIS had an α coefficient of 0.734, demonstrating acceptable reliability. The PSQI, ISI, and AIS showed outstanding diagnostic ability with an AUC of 0.960 (95% CI: 0.946, 0.974), 0.911 (95% CI: 0.882, 0.941), and 0.876 (95% CI:0.837, 0.916). The best diagnostic cutoffs for PSQI, ISI, and AIS are ≥9, ≥15, and ≥8.

**Conclusion:**

Each of the three questionnaires has advantages and disadvantages when assessing insomnia. In the evaluation of insomnia in IS patients, the best questionnaire selection should be made according to the purpose of clinical evaluation and considering the sensitivity and specificity.

## 1. Introduction

Stroke is a leading cause of death and disability around the world (1). Ischemic stroke (IS) has high rates of morbidity, disability, recurrence and mortality (2). Insomnia is one of the common complaints of IS survivors. In contrast to other long-term sequelae of IS such as mobility and cognitive impairment, insomnia has received less attention and related research, despite being a risk factor for stroke (3). In accordance with the latest manual by the American Association of Sleep Medicine (AASM), insomnia is defined as persistent sleep problems and daytime socio-occupational dysfunction, which may be actual or perceived despite adequate sleep opportunities (4). According to the International classification of sleep disorders-3 edition (DSM-3) and the Chinese expert consensus on the assessment and management of stroke related sleep disorders (CEC-SSD) (5), stroke-related insomnia consists of two conditions: (i) post-stroke insomnia: insomnia first appears after stroke; (ii) stroke with insomnia: insomnia existing before stroke persists or worsens after stroke and meets the diagnostic criteria for insomnia. According to reports published 30 ~ 68% of poststroke patients were burdened with insomnia (6–8). In addition, insomnia is also thought to increase the risk of psychological problems (depression and anxiety), physical function (disability), and cognitive function (dementia) in IS patients (9, 10).

Strokes are more likely to occur in people who suffer from insomnia. The results of a meta-analysis of 160,867 patients in 15 studies showed that falling/maintaining asleep difficulty, and non-restorative sleep were positively strongly associated with the risk of stroke (11). According to a prospective clinical cohort study of stroke patients, insomnia patients were more likely to be depressed, anxious, disabled, and have difficulty returning to work than stroke patients without insomnia after 1 year after stroke (12). Surveys such as that conducted by Huang et al. (13) have shown that a negatively association between insomnia and the improvement in activities of daily living was found in subacute stroke inpatients. Tang et al. (14) showed that insomnia may make stroke survivors more susceptible to suicide. Kim et al. (10) demonstrated that insomnia had a negative effect on quality of life in stroke patients at the initial phases of rehabilitation. Also, insomnia IS patients have a higher recurrence stroke rate in the first year and a higher mortality rate within 6 years compared with no-insomnia IS patients (15).

Although the prevalence of insomnia continues to rise in many countries around the world (16, 17), patients often treat insomnia as a lifestyle issue rather than a major health problem (18). Also, the importance of insomnia diagnosis has been neglected in the routine medical examination of some IS patients. Clinician assessment and judgment based on the DSM-5 and CEC-SSD remain the criteria for diagnosing insomnia in stroke patients. Polysomnography (PSG) is considered as a common objective measure for the measurement of sleep disorders. However, PSG is not easy to obtain for most clinicians’ daily insomnia diagnosis routine (19), and is mostly used for the diagnosis of sleep disordered breathing (SDB). At the same time, PSG is very expensive for epidemiology and research, takes a long time and is not in line with clinical practice (19). However, multiple insomnia questionnaires have been developed including the PSQI, ISI, and AIS. All three questionnaires were used multiple times and translated into multiple languages and have been widely used in China. These questionnaires are considered to be efficient screening tools for insomnia. They are simple to perform and cost effective and do not demand additional special apparatus or facilities. The use of brief questionnaires for subjective assessment saves time and effort and ensures a high level of patient subjective willingness and compliance. Also, due to the self-explanatory nature of these tools, the need for and reliance on specialists and clinicians can be significantly reduced (20, 21). The three insomnia assessment tools used in this study, PSQI, ISI, and AIS, were not developed specifically for IS patients, and each scale validation was based on primary sleep disorders patients (22). Because of the significant role that the rating scales plays in the diagnosis of insomnia in IS patients, psychometric properties, cut-off values and diagnosis effectiveness of these tools need to be evaluated in IS patients. The purpose of this study was to the establish reliability and determine the optimal threshold values of PSQI, ISI, and AIS in the diagnosis of insomnia of IS patients.

## 2. Methods

### 2.1. Participants

This study recruited 569 IS patients on the inpatient medical care rosters of three general hospitals in Shanghai from January 2021 to September 2021. The three hospitals recruited 186, 165 and 218 IS patients, respectively. All patients received neuroradiological exam and the IS diagnosis were consistent with the “Chinese guidelines for diagnosis and treatment of acute ischemic stroke 2018 (23).” The following conditions were excluded:

unable to respond appropriately to questionnaires (such as aphasia);
transient ischemic attack (TIA) diagnosed by neurologists;
diagnosed with dementia, or another neurodegenerative or neurological condition.

The diagnosis of stroke-related insomnia (SRI) was determined by neurologist after evaluation and needed to meet both the diagnosis of stroke and insomnia. According to DSM-5 and CEC-SSD (5), the diagnosis of insomnia in this study was as follows. Patients who met all the following five conditions were insomnia IS patients, and the others were no-insomnia IS patients:

Patients complain of disgruntlement over sleep quality or quantity. They have one or more of the following symptoms: difficulty falling or maintaining sleep; wake up early and are unable to fall back asleep.
Patients complain that sleep disorders make them feel clinically significant distress or make it hard for them to do important things like socialize, work, behavioral, or other significant functions. They have one or more of the following symptoms: fatigue or lack of energy; decreased attention/concentration/memory; emotional instability; daytime fatigue; behavioral problems (a tendency to be hyperactive, impulsive, or aggressive); lack of energy or motivation; concerns about the quality of sleep.
These abnormalities cannot be accounted by inadequate sleep opportunities or inferior sleep environment.
Sleep disturbances and daytime symptoms ≥3 times a week.
The above symptoms are not explained by other sleep disorders.

In order to prevent the impacts of unstable neurological circumstances and environmental changes on the results, our investigation was conducted when the patient was conscious and exhibited stable vital signs after routine neurology treatment. The questionnaires of basic patient information, neurological function, three sleep assessment were applied at a mean of 5.79 days (*SD* = 2.34) after admission to the hospital. All 569 patients included in the study completed basic information collection and insomnia assessment using three scales (PSQI, ISI, AIS). The investigators conducted face-to-face interviews with IS patients at the neurology ward using PSQI, ISI, and AIS questionnaires. All questionnaires were evaluated when patients fully understood the contents of the questionnaire items.

Ethical approval was obtained from the Ethics Approval Committee of Shanghai Tenth People’s Hospital (Approval No. SHSY-IEC-KY-4.0/17–47/01). All participants agreed to participate in this study.

### 2.2. Measures and questionnaires

#### 2.2.1. Basic information collection and functional assessment

The basic information of patients included age, sex, body mass index (BMI), marital status, education years, occupation, smoking, alcohol consumption, hypertension, diabetes, coronary heart disease. In this study, “smoke” was operationally defined as “current smokers” who had smoked within 30 days before the survey. “Drink” was operationally defined as consuming more than 15 g of alcohol per day within 30 days before the survey.

Each patient was assessed on the National Institute of Health stroke scale (NIHSS). The NIHSS was used to assess the severity of cerebral infarction and the degree of neurological deficit in patients. High NIHSS scores indicate more severe cerebral infarction, resulting in greater disability and functional decline. Also, each patient was assessed for activities of daily living (ADL) using Barthel index (BI). ADL competence is one of the most important indicators of the effectiveness of rehabilitation (24). The Barthel index (BI) is the most commonly used scale in the world to assess ADL competence (25). The BI consists of 10 items: feeding, bed and wheelchair transfer, personal hygiene, toileting, bathing, walking, walking up and down stairs, dressing, bowel control and urinary control. It is mainly suitable for detecting the changes of independent living activities of the elderly and patients before and after treatment.

#### 2.2.2. PSQI

The PSQI has been extensively used in China to measure insomnia symptoms as an essential measure that is recommended globally (26). Based on DSM-5 and CEC-SSD, in clinical patients, the PSQI is the most widely used subjective measure of sleep dysfunction (27). The PSQI is a self-rating questionnaire, composed of 19 self-rated items (0 ~ 3 scale) with a total score from 0 to 21 assessing 7 domains of sleep quality and sleep disorders during the course of the past month: (i) subjective sleep quality (C1), (ii) sleep latency (C2), (iii) sleep duration (C3), (iv) normal sleep efficiency (C4), (v) sleep disturbances (C5), (vi) the application of sleep medication (C6), and (vii) daytime dysfunction (C7). The higher score indicates the worse sleep quality (28). A considerable amount of literature has been published on optimal threshold of PSQI in determining good or bad sleep quality. PSQI has not only been widely used in non-clinical populations, but has also been studied in a variety of clinical populations, including post-surgical patients (29), hemodialysis patients (30), schizophrenia (31), and anxiety (32). The cut-off values for the diagnosis of insomnia differ for different diseases. A cutoff score of 5 was suggested by Tsai et al. (33) to distinguish primary insomnia with Chinese version of PSQI among community-dwelling adults. A cross-sectional study of 327 IS patients suggested 8 as the cut-off score (34). The PSQI scale used in this study is the Chinese version translated by Liu et al. (35) in 1996. When the total PSQI score over 7, the sensitivity of distinguishing between normal and sleep-disordered populations was 98.3% and the specificity was 90.2% (35). The time to complete the PSQI assessment is approximately 5 to 10 min.

#### 2.2.3. ISI

The ISI is a reliable instrument for population-level detection of insomnia cases and is sensitive to treatment response in clinical patients (36). The ISI is a seven-item, subjective assessment and screening tool that primarily assesses the character, gravity, and influence of insomnia over the previous 4 weeks (37). The items evaluate (i) difficulty falling asleep (initial) (I1a), (ii) difficulty maintaining sleep (middle) (I1b), (iii) early awakening (terminal) (I1c), (iv) satisfaction with current sleep situation (I2), (v) the degree of hindrance with routine function caused by sleep dilemmas (I3), (vi) the degree of impact or impairment on quality of life caused by the sleep difficulty (I4), and (vii) level of anxiety attributed to the sleep predicament (I5) (38). The time to complete the ISI assessment is approximately 5 min. The ISI uses a Likert scale of five points (0 ~ 4 scale), with a total score between 0 and 28. According to Reinsel (39), the best clinical cutoff score for identifying sleep disorders in breast cancer patients is 8. A cutoff score of 8 was suggested by Savard et al. (40) for identifying sleep difficulties in cancer patients, which produced 94.7% sensitivity and 47.4% specificity in cancer patients according to the DSM-IV criteria. At present, ISI has not established a cutoff score for the diagnosis of insomnia in many clinical patients (22).

#### 2.2.4. AIS

The AIS was developed as a standard sleep assessment tool to quantify the degree of sleep difficulty, in accordance with the International Classification of Diseases-10 edition (ICD-10) (41). There are eight items: the first five are concerned with (i) time needed to fall asleep (A1), (ii) wakening during the night (A2), (iii) waking up earlier than desired time (A3), (iv) sleep duration (A4), and (v) sleep quality (A5); while the last three items concerned with (vi) daytime emotional state (A6), (vii) daytime functioning capacity (A7), and (viii) daytime sleepiness (A8). The AIS uses a Likert scale of 4 points (0 = no problem; 3 = serious problem), with an overall score from 0 to 24. Higher AIS scores represent higher levels of insomnia. The AIS has been translated into numerous languages and has been used by various populations worldwide, including English (41), Chinese (42), and Spanish (43). With a cut-off score of 6, AIS can be used for insomnia screening with the sensitivity of 93% and the specificity of 85% in Taiwanese cancer patients (42). A score of 6 was found to be the optimal cutoff when logistic regression analysis was performed between the total AIS score and ICD-10 insomnia diagnosis (44). Jeong et al. (45) also suggested the commonly accepted cut-off to be 6. In clinical practice and research, AIS can be utilized as a screening instrument to measure the severity of sleep-related issues as well as a tool in reliable insomnia screening. Although AIS is regarded as a useful insomnia screening tool for research prediction, it has not been tested on the IS patients.

### 2.3. Data analysis

EpiData 3.0 was used for data entry and aggregation for IS patients, and two researchers completed data input separately to ensure data accuracy. The IBM SPSS 22.0 and MedCalc 21.0 were used to implement statistical analysis. Kolmogorov–Smirnov test was used to test the normality of the data distribution, with *p* > 0.05 considered as normal distribution. Descriptive statistics were reported as mean ± standard deviation (SD) for variables with normal distributions and as median (interquartile range, IQR) variables with for skewed distributions. If the variables in each sample were normally distributed and the overall variance was equal, ANOVA was used to compare the means between the insomnia group and the non-insomnia group, and nonparametric test of Mann–Whitney *U* test was used if not. Chi-square test was used to compare the rates of categorical variables between the two groups.

ROC curve analysis was used to analyze the diagnostic validity of PSQI, ISI, and AIS. The area under ROC curve (AUC) was performed to evaluate the accuracy of PSQI, ISI, and AIS for the diagnosis of insomnia in IS patients. AUC is currently accepted as a diagnostic evaluation index and a method to determine the optimal diagnostic cutoff values. The value of AUC ranges from 0.5 to 1. The diagnostic value is acceptable when the score is between 0.71 and 0.8, excellent when it is 0.81–0.9, and outstanding when it is >0.9 (46). DeLong’s test (47) was performed to compare multiple ROC curves. Youden index was performed to determine the optimum cutoff diagnostic values of PSQI, ISI, and AIS (48). Cronbach’s α and McDonalds Ω coefficient was used for the reliability analysis of this study. The sensitivity, specificity, positive likelihood ratio (PLR), negative likelihood ratio (NLR), and diagnostic validity of the three scales were also analyzed. Cohen’s Kappa consistency was performed between insomnia diagnosis based on PSQI, ISI, and AIS and clinician-based insomnia diagnosis.

## 3. Results

### 3.1. Characteristics of IS patients

The Demographics of IS patients in the insomnia group and no-insomnia group in this study are shown in Table 1. A total of 569 patients were separated into insomnia group (*n* = 89) and no-insomnia group (*n* = 480) in accordance with the diagnostic criteria of insomnia in IS patients. In this study, 337 (59.23%) patients were male and 232 (40.77%) were female. The average age was 63.25 (*SD* = 10.34) and the average BMI was 24.27 (*SD* = 2.92). Additionally, 157 patients (27.59%) were employed before stroke. The median (IQR) of NIHSS and ADL scores of IS patients were 3 (3) and 55 (25). 415 (72.93%) IS patients have hypertension and 251 (44.11%) IS patients have diabetes. A comparative analysis of the insomnia group and the no-insomnia group revealed significant differences in terms of age, sex, BMI, and drinking. When the insomnia group was compared with the non-insomnia group, it was found that the insomnia group was older and included more female participants. Meanwhile, patients in the insomnia group had higher BMI and more alcohol consumption.

**Table 1:** Demographics of IS patients in the insomnia group and no-insomnia group (*n* = 569).

Variables	No-insomnia group (*n* = 480)	Insomnia group (*n* = 89)	*X*^2^/*F*/*Z*	*p*
Age (years), mean ± SD	62.72 ± 10.57	66.13 ± 8.54	8.278	0.004
Sex, n (%)			26.000	<0.001
Female	174 (36.3)	58 (65.2)		
Male	306 (63.8)	31 (34.8)		
BMI (Kg/m²), mean ± SD	24.12 ± 2.84	25.09 ± 3.21	8.485	0.004
Marital status, n (%)			0.145	0.703
Married	442 (92.1)	83 (93.3)		
Unmarried/Divorced/Widowed	38 (7.9)	6 (6.7)		
Education, n (%)			0.490	0.484
≤9 y	186 (38.8)	38 (42.7)		
>9 y	294 (61.3)	51 (57.3)		
Occupation, n (%)			3.320	0.068
Retired	340 (70.8)	72 (80.9)		
Employed	140 (29.2)	17 (19.1)		
Drink, n (%)	241 (50.2)	29 (32.6)	9.352	0.002
Smoke, n (%)	124 (25.9)	28 (31.5)	1.165	0.280
Hypertension, n (%)	353 (73.5)	62 (69.7)	0.572	0.449
Diabetes, n (%)	220 (45.8)	31 (34.8)	3.686	0.055
Coronary heart disease, n (%)	47 (9.8)	11 (12.4)	0.541	0.462
NIHSS (M, IQR)	3 (3)	3 (3)	−0.309	0.756
BI (M, IQR)	55 (25)	55 (30)	−0.004	0.997

SD, Standard deviation; n, number of patients; M, IQR, Median, Inter Quartile Range; BMI, Body mass index; NIHSS, National Institute of Health stroke scale; BI, Barthel index.

### 3.2. Analysis of PSQI, ISI, and AIS scores

The number of IS patients’ responses of the three questionnaires in the insomnia and no-insomnia groups is shown in Table 2. The median (IQR) of PSQI score in no-insomnia group was 3(2), and median (IQR) of PSQI score in insomnia group was 11(1). The median (IQR) of ISI score in no-insomnia group was 13(6), and median (IQR) of ISI score in insomnia group was 17(3). The median (IQR) of AIS score in no-insomnia group was 5(3), and median (IQR) of AIS score in insomnia group was 9(5). The results of the Mann–Whitney U test showed there were significant differences in scores of PSQI, ISI, and AIS between the insomnia group and the no-insomnia group (*p* < 0.001).

**Table 2:** The scores of PSQI, ISI, and AIS for insomnia group and no-insomnia group (*N* = 569).

Variables	No-insomnia group (*n* = 480)	Insomnia group (*n* = 89)	*p*
PSQI, (M, IQR)	3 (2)	11 (1)	<0.001
ISI, (M, IQR)	13 (6)	17 (3)	<0.001
AIS, (M, IQR)	5 (3)	9 (5)	<0.001

M, IQR, median, interquartile range.

### 3.3. Reliability of the PSQI, ISI, and AIS

The PSQI and ISI showed high reliability with Cronbach’s α-values of 0.875 and 0.858, while the AIS had an α coefficient of 0.734, demonstrating acceptable reliability. The factor analysis demonstrated that the ISI and AIS scale both emerged as a sole component (37, 41). The PSQI and ISI showed high reliability with McDonalds Ω values of 0.895 and 0.864, while the AIS had an α coefficient of 0.736, which is consistent with the Cronbach’s alpha coefficient.

### 3.4. Diagnostic validity of PSQI, ISI, and AIS

**Figure 1:**
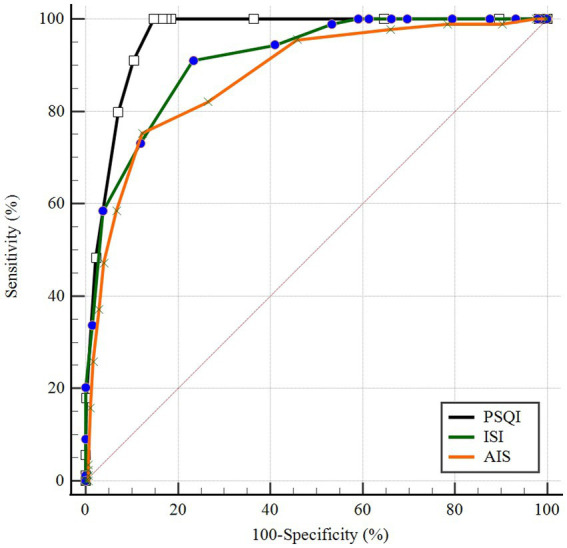
ROC curves of PSQI, ISI, and AIS in IS patients.

**Figure 2:**
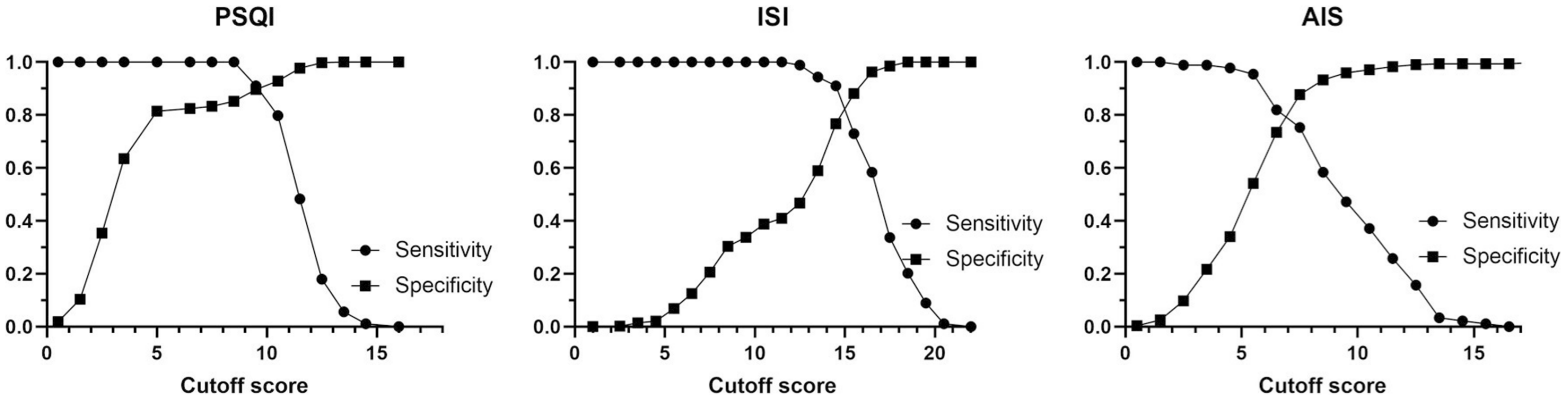
Sensitivity and specificity of PSQI, ISI, and AIS for various cutoff scores.

ROC curves of PSQI, ISI, and AIS in IS patients were showed in Figure 1. The AUCs of PSQI and ISI were 0.960 (95% CI: 0.946, 0.974) and 0.911 (95% CI: 0.882, 0.941), which were greater than 0.9, showing outstanding diagnostic ability of insomnia in IS patients. The AUC of AIS was 0.876 (95% CI: 0.837, 0.916), which ranged from 0.8 to 0.9, showing excellent diagnostic ability with PSQI and ISI in IS patients. DeLong’s test indicated that PSQI can be considered to have better diagnostic validity than ISI and IAS, while ISI and AIS cannot be differentiated regarding diagnostic validity (Table 3). The current study compared different PSQI, ISI, and AIS cutoff scores and the resulting sensitivity and specificity are shown in Figure 2. After determining the best cutoff values based on Youden’s index, which were 9 for PSQI, 15 for ISI and 8 for AIS, other statistics were further identified, such as sensitivity, specificity, Youden index, positive and negative likelihood ratio (Table 4). When it came to distinguishing between patients with and without insomnia, the PSQI had the highest sensitivity.

**Table 3:** DeLong’s test on pairwise comparison of ROC curves.

Variables	PSQI and ISI	PSQI and AIS	ISI and AIS
Difference between areas	0.049	0.084	0.0348
Standard error	0.0149	0.0201	0.0234
95% *CI*	(0.019, 0.079)	(0.044, 0.123)	(−0.011, 0.081)
Z statistic	3.281	4.164	1.488
*p*	0.001	<0.0001	0.1368

**Table 4:** Accuracy and diagnostic validity of PSQI, ISI, and AIS in IS patients.

Variables	PSQI	ISI	AIS
AUC (*95%CI*)	0.960 (0.946, 0.974)	0.911 (0.882, 0.941)	0.876 (0.837, 0.916)
Cutoff point	≥9	≥15	≥8
Se	100%	91.01%	75.28%
Sp	85.21%	76.67%	87.71%
YI	0.852	0.677	0.630
PLR	6.761	3.900	6.125
NLR	0.000	0.117	0.282
Cohen’s Kappa	0.643 (0.570, 0.716)	0.459 (0.385, 0.533)	0.539 (0.451, 0.627)

AUC, Area Under Curve; Se, Sensitivity; Sp, Specificity; YI, Youden index; PLR, Positive likelihood ratio; NLR, Negative likelihood ratio.

Cohen's Kappa = agreement with clinician-based diagnosis.

Cohen’s Kappa test was used to compare the consistency between clinician-based insomnia diagnosis and questionnaire-based insomnia diagnosis in Table 4. In the questionnaire-based insomnia diagnosis, PSQI ≥9, ISI ≥ 15 and AIS ≥ 8 was considered as insomnia. Comparing PSQI with the clinician-based insomnia diagnosis revealed that Cohen’s kappa was 0.643 (95% CI: 0.570, 0.716). However, comparing ISI and AIS with insomnia diagnosis revealed that Cohen’s kappa was 0.459 (95% CI: 0.385, 0.533) and 0.539 (95% CI: 0.451, 0.627). PSQI can be considered to have more diagnostic concordance with clinicians than ISI (no CI overlap) while AIS has the same concordance as both PSQI and ISI (CI overlap).

## 4. Discussion

Insomnia is a common psychiatric complaint in IS patients (9). The reported prevalence of insomnia varies widely among studies due to the different definitions and assessment tools used. Among the 569 IS patients contained in this study, 89 were diagnosed as insomnia by clinicians. The prevalence of pre-stroke insomnia in IS patients was found to be 15.64% in this study, lower than that of previously reported levels. Surveys such as that conducted by Leppävuori et al. (49) have shown that 38.6% had insomnia before the stroke and 18.1% had insomnia after the stroke. Another study found a 12% rate of new-onset insomnia after stroke when excluding patients who had insomnia before stroke (50). A study from China found that the prevalence of insomnia in stroke patients was 57.9%, of which 32.2% had insomnia before the stroke and 25.7% had new insomnia after the stroke (51). A 4-year follow-up study of 21,438 insomniacs and 64,314 non-insomniacs found that the incidence of stroke was significantly higher in insomniacs than in non-insomniacs (incidence rate ratio = 1.85; 95% CI: 0.7, 2.05) and those insomniacs had 54% higher risk of developing stroke compared with non-insomniacs (adjusted hazard ratio = 1.54; 95% CI: 1.38, 1.72) (3). There is mounting clinical evidence to support a bidirectional relationship between insomnia and stroke. Insomnia is likely to be an independent risk factor for stroke, and stroke may also be a causative factor for the development of insomnia. Insomnia should be given more attention in IS patients.

This study demonstrated high sensitivity and specificity of the PSQI, ISI, and AIS in screening for insomnia in IS patients. It was discovered that the PSQI scale was the most sensitive (identifying insomnia in 100% of the IS patients when the cutoff value is 9), while DeLong’s test indicated that ISI and ASI were less but similarly sensitive, with ISI identifying insomnia in 91.01% of the IS patients when the cutoff value is 15, and AIS identifying insomnia in 75.28% of the IS patients when the cutoff value is 8. Some studies suggest that the diagnostic sensitivity to distinguish good sleep from poor sleep was 89.6% and the specificity was 86.5% when the total PSQI score > 5 (52). The Youden index of the PSQI was 0.852, with a strong ability to screen insomnia patients from non-insomnia patients. When the effect of morbidity was excluded, the positive likelihood ratio (PLR) for the PSQI was 6.761, which is a higher than ISI and AIS. Also, according to Charles, the ISI cutoff of 10 was the optimal score for identifying insomnia cases in the community people, with an 86.1% sensitivity and 87.7% specificity (36). When diagnosing insomnia on a score of ≥6, the AIS scale had a sensitivity of 93%, and a specificity of 85%, and the overall correct case identification rate is 90% (44). This confirmed the findings of these scales have excellent performance of these scales in screening for insomnia in IS patients in the literature (34, 36, 42). Our analysis showed that, identical to the widely used cutoff values for insomnia diagnosis in the general population in China, the best cutoff values in screening for insomnia in IS patients of the ISI questionnaires were “15.” However, the best cutoff values in screening for insomnia in IS patients of the PSQI and AIS questionnaires were “9” and “8” respectively, higher than the widely used diagnostic cutoff values for insomnia population in China.

Cohen’s kappa consistency statistic check was used to analyze the consistency of PSQI, ISI, and AIS with clinician-based insomnia diagnostic in IS patients. The kappa coefficient of PSQI, ISI, and AIS were 0.643, 0.459, and 0.539 and the asymptotic 95% confidence interval were (0.570, 0.716), (0.385, 0.533), and (0.451, 0.627). Landis and Koch (53) proposed that a kappa in the range of 0.41 ~ 0.60 be considered “moderate” agreement, kappa = 0.61 ~ 0.80 be considered “substantial” agreement, and kappa >0.81 be considered “almost perfect” agreement. This indicates that the consistency between PSQI with clinician-based insomnia diagnostic were substantial consistent. The consistency between ISI and AIS with clinician--based insomnia diagnostic was moderately consistent.

The PSQI focuses on the sleep status of patients in the past 4 weeks. Also, there are four types of responses (“not during the last month,” “less than once a week,” “once or twice a week,” “three or more times per week”) instead of using “very poor,” “poor,” “good,” “very good” and similar language to assess sleep. Such descriptions are important to accurately measure the severity of the IS patient’s sleep-related components. We believe this could help our patients better understand the PSQI question and choose the option that best matches their situation, compared to the AIS and ISI. In addition, these descriptions and response category of sleep quality severity can be translated into numerical rating ranges, so this may explain the greater sensitivity of PSQI in assessing insomnia in IS patients. PSQI is a widely used sleep quality evaluation tool to measure sleep quality and disturbances over the prior month and to discriminate between “good” and “poor” sleepers (22). Meanwhile, PSQI is often used together with the Epworth Sleepiness Scale (ESS) to assess daytime sleepiness, such as in medical students (54), COPD and asthma (55), or women with premenstrual syndrome (56).

Although the ROC curve was used to evaluate the diagnostic performance of the three questionnaires in insomnia, it was not the only thing to consider in choosing an assessment tool. The three questionnaires have similar content and language descriptions of sleep problems. Each of the three questionnaires has advantages and disadvantages when assessing insomnia. The PSQI cannot collect information about the patient’s need of treatment options and other aspects. The PSQI scale has more questions and takes longer to assess compared to the ISI and AIS, so it performs poorly in rapidly assessing the severity of insomnia in clinical patients. It should be emphasized that PSQI is the only one of the three questionnaires that needs to calculate and transform scores. Because of the need to integrate various responses and calculate such variables as sleep efficiency, hand-calculation of scores may be somewhat burdensome (22). This undoubtedly caused additional inconvenience and time consumption for clinical evaluation.

One advantage of ISI is that there are data based on which insomnia can be classified hierarchically. In a study which set out to investigate insomnia in long-term hospitalized older adults, Aluzaite et al. (57) classified ISI scores as follows: 0 ~ 7 points represent no insomnia; 8 ~ 14 points represent sub-threshold insomnia; 15 ~ 21 points represent moderate insomnia; 22 ~ 28 points represent severe insomnia. Under this score classification, the degree of insomnia in patients can be measured. Meanwhile, for PSQI and AIS such thresholds to measure the severity of insomnia in patients have not yet been investigated. In many clinical assessments, PSQI is often used as an index to measure the overall sleep quality, and ISI is used to measure the severity of insomnia (58). ISI focuses on the subjective symptoms of insomnia, the consequences and the degree of distress, and is sensitive to detect changes in sleep conditions brought about by treatment. However, researchers need to be alert to the possibility of false positive results when patients subjectively amplify their insomnia feelings.

The AIS is designed to assessment subjective feeling of sleep difficulty that only the subjective feelings of the interviewee are considered for the rating (41). In addition, the severity of the patient’s sleep difficulty as estimated by the interviewer, such as the hours of sleep or the approximate duration of sleep onset latency (41), was not considered in the AIS score since the interviewer’s judgment may not be consistent with the patient’s subjective assessment. Patients may exaggerate the negative feelings caused by short sleep duration, leading to bias in AIS assessment, which may also be the reason for the lower sensitivity of AIS.

There are several limitations to this study: All subjects were not tested with Polysomnography in this study. We compared the application of the three questionnaires in the diagnosis of insomnia, but did not give grade judgments for the severity of insomnia. Finally, because the information was gathered based on self-reporting, the data may be prone to information bias.

## 5. Conclusion

This study demonstrated high sensitivity and specificity and excellent diagnostic ability of the PSQI, ISI, and AIS questionnaires in screening for insomnia in IS patients. We found the best diagnostic cutoffs for PSQI, ISI, and AIS are ≥9, ≥15, and ≥8, respectively. Based on these cutoff values, the PSQI scale was revealed to be the most sensitive, while the ISI was the moderate sensitive and AIS was the least sensitive. Each of the three questionnaires has advantages and disadvantages when assessing insomnia. In the evaluation of insomnia in IS patients, the best questionnaire selection should be made according to the purpose of clinical evaluation (such as screening potential insomnia patients or the diagnosis of clinical insomnia) and considering the sensitivity and specificity of the questionnaire.

## Data availability statement

The raw data supporting the conclusions of this article will be made available by the authors, without undue reservation.

## Ethics statement

The studies involving human participants were reviewed and approved by the Institutional Review Boards of the Tenth People’s Hospital, Tongji University (Approval No. SHSY-IEC-KY-4.0/17–47/01). The patients/participants provided their written informed consent to participate in this study.

## Author contributions

SN and YS: study conception and design, manuscript drafts and revisions. SN, SD, LCW, and LW: data collection. SN and QW: data analysis. SN, QW, SD, LCW, and YS: agree with manuscript results and conclusions. All authors approved the final manuscript and act as guarantors for the study.

## Funding

This research was supported by the grants from the general project of National Natural Science Foundation of China (71774117).

## Conflict of interest

The authors declare that the research was conducted in the absence of any commercial or financial relationships that could be construed as a potential conflict of interest.

## Publisher’s note

All claims expressed in this article are solely those of the authors and do not necessarily represent those of their affiliated organizations, or those of the publisher, the editors and the reviewers. Any product that may be evaluated in this article, or claim that may be made by its manufacturer, is not guaranteed or endorsed by the publisher.
